# Supplementary figures and images for: Hyperosmotic stress induces epithelial-mesenchymal transition through rearrangements of focal adhesions in tubular epithelial cells
Source: PLoS One. 2021 Dec 21;16(12):e0261345. doi: 10.1371/journal.pone.0261345 (PMC8691603; doi:10.1371/journal.pone.0261345)

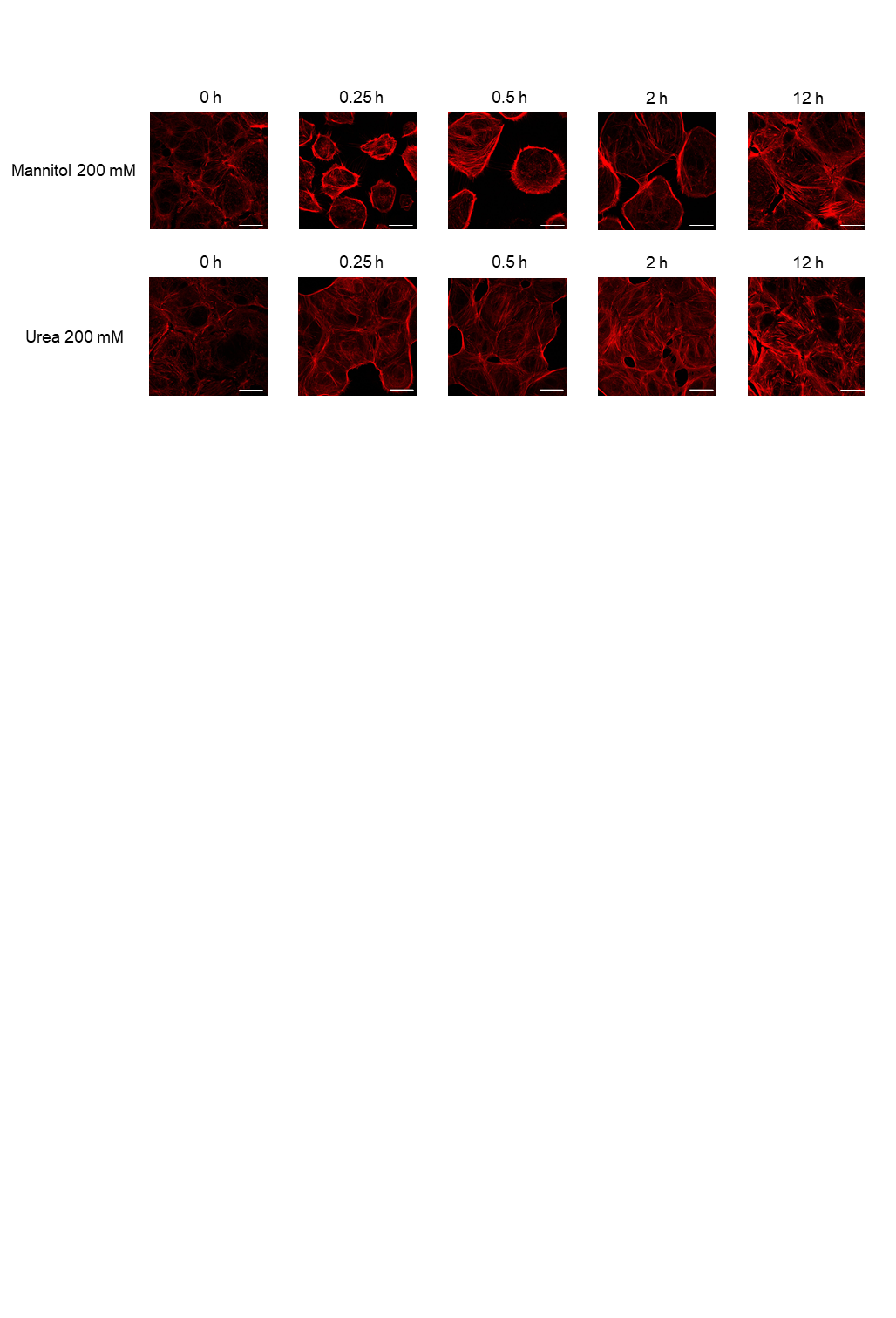

Supplement: S1 Fig — Cells were cultured with 200 mM mannitol or urea for 0, 0.25, 0.5, 2, and 12 h. Typical fluorescence images of F-actin. Bar, 25 μm. (TIF) [file pone.0261345.s001.tif]

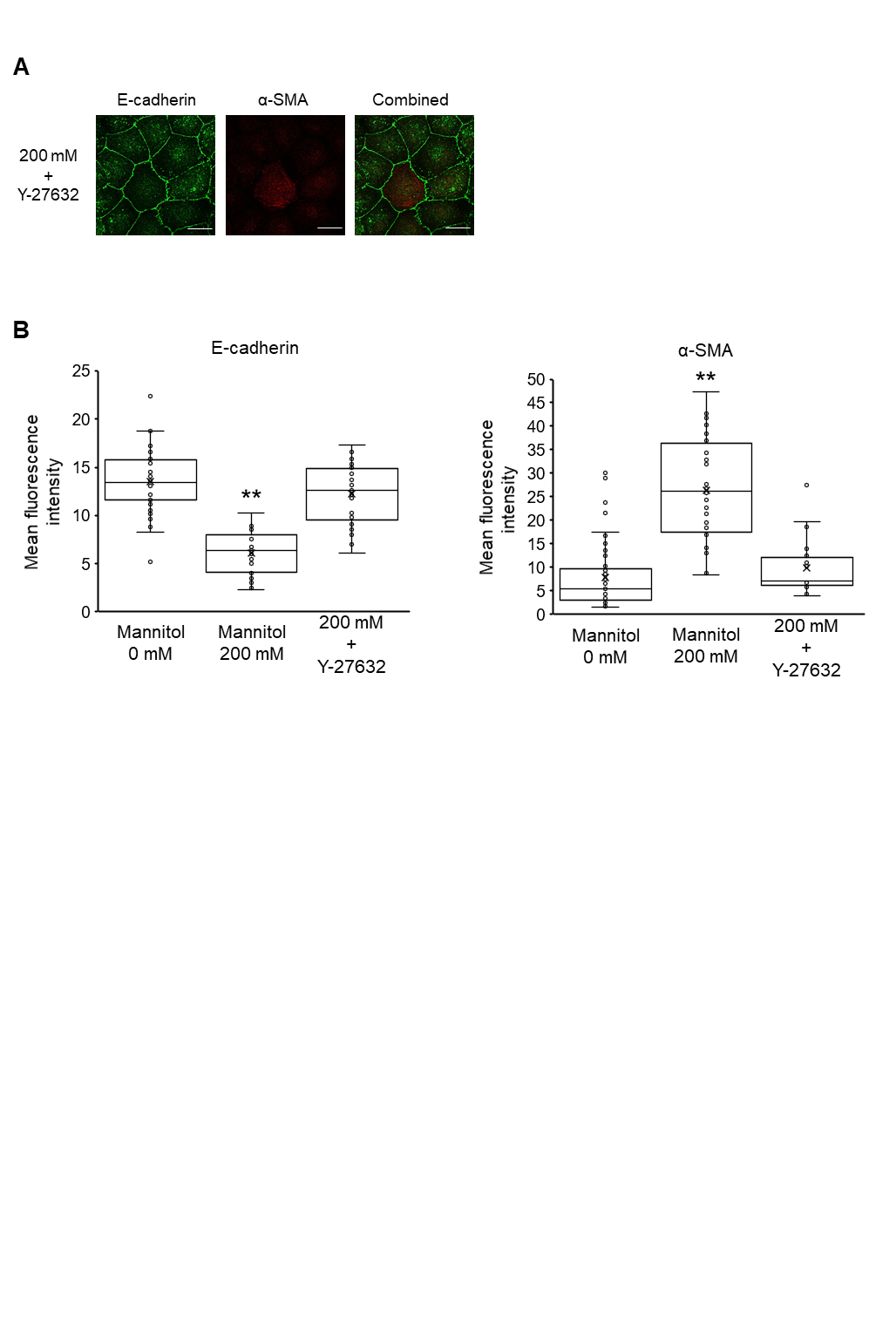

Supplement: S2 Fig — (A) Typical fluorescence images of E-cadherin (green), α-SMA (red), and combined (green and red) cotreated with mannitol (200 mM) and Y-27632 (1 μM) for 12 h. Bar, 25 μm. (B) Quantitation of the changes in the mean fluorescence intensity of E-cadherin and α-SMA (n = 34 from 200 mM + Y-27632) by immunofluorescence staining. The data of mannitol (0 and 200 mM) were identical to those in Fig 1B and 1C, which were shown for comparisons. Data are presented as box and whisker plots with average (×), median, IQR, and minimum and maximum values. The n indicates the number of cells analyzed. **P < 0.01 from the data of 0 mM (Tukey’s test). (TIF) [file pone.0261345.s002.tif]

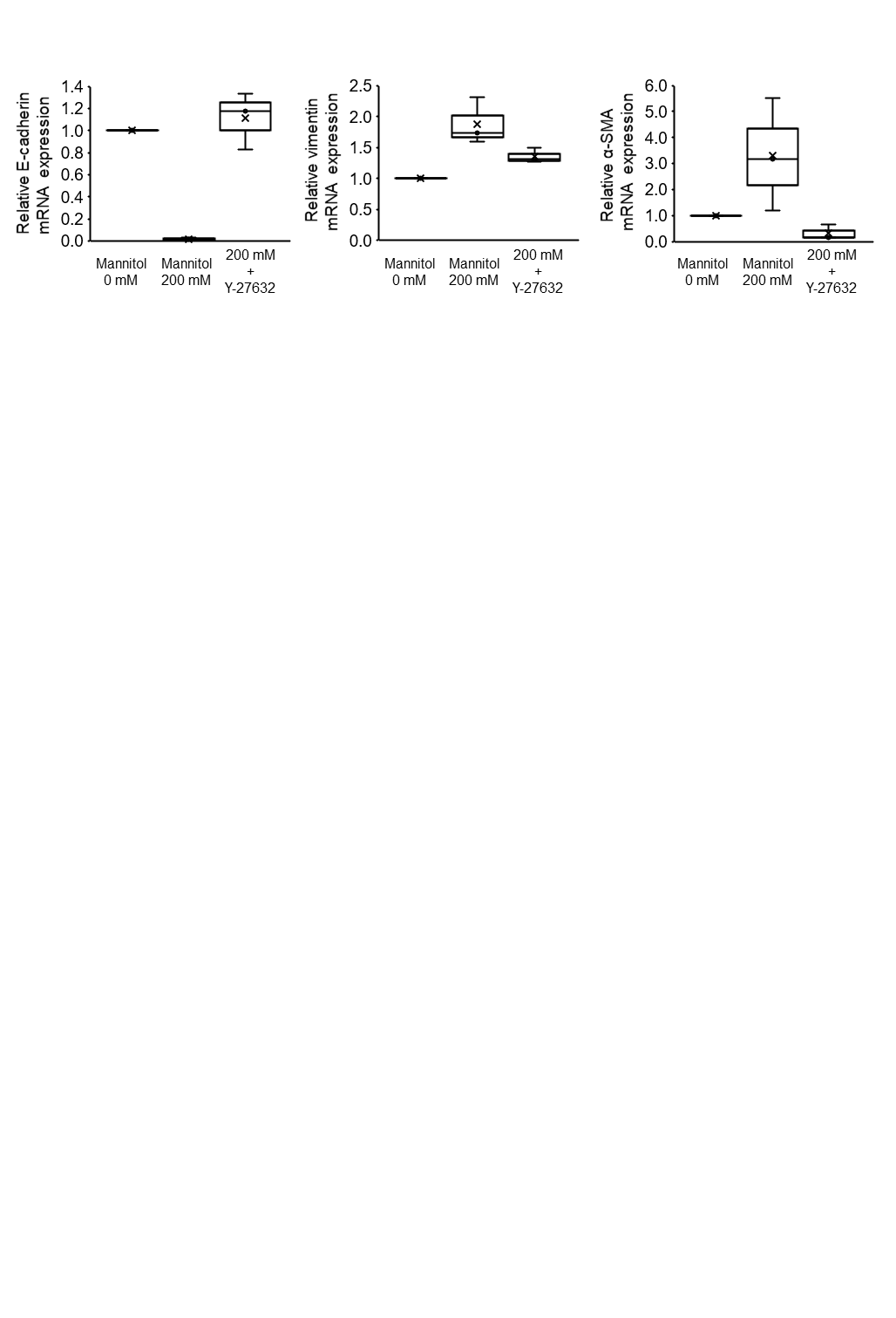

Supplement: S3 Fig — NRK-52E cells were cotreated with mannitol (200 mM) and Y-27632 (1 μM), and mRNA expression was analyzed by real-time PCR. Quantitation of the changes in E-cadherin (n = 3 from 0 mM, n = 3 from 200 mM, n = 3 from 200 mM + Y-27632), vimentin (n = 3 from 0 mM, n = 3 from 200 mM, n = 3 from 200 mM + Y-27632) and α-SMA (n = 3 from 0 mM, n = 3 from 200 mM, n = 3 from 200 mM + Y-27632). Relative gene expression levels were calculated considering mannitol (0 mM) as 1 and plotted. Data are presented as box and whisker plots with average (×), median, IQR and minimum and maximum values. The n indicates the number of independent experiments. (TIF) [file pone.0261345.s003.tif]
