# Supplementary material for: Hyperosmotic stress induces epithelial-mesenchymal transition through rearrangements of focal adhesions in tubular epithelial cells
Source: PLoS One. 2021 Dec 21;16(12):e0261345. doi: 10.1371/journal.pone.0261345 (PMC8691603; doi:10.1371/journal.pone.0261345)
Supplement: S1 Raw images — Raw images of western blot using in the Fig 6A. (PDF) [file pone.0261345.s004.pdf]

# S1 raw images

**Original blots from Fig 6A (α-SMA and GAPDH)**
NRK-52E cells were treated with mannitol (200 mM) or cotreated with Y- 27632 (1 μM) for 12 h.

The band corresponding to α-SMA is indicated by the arrow.

42 kDa →

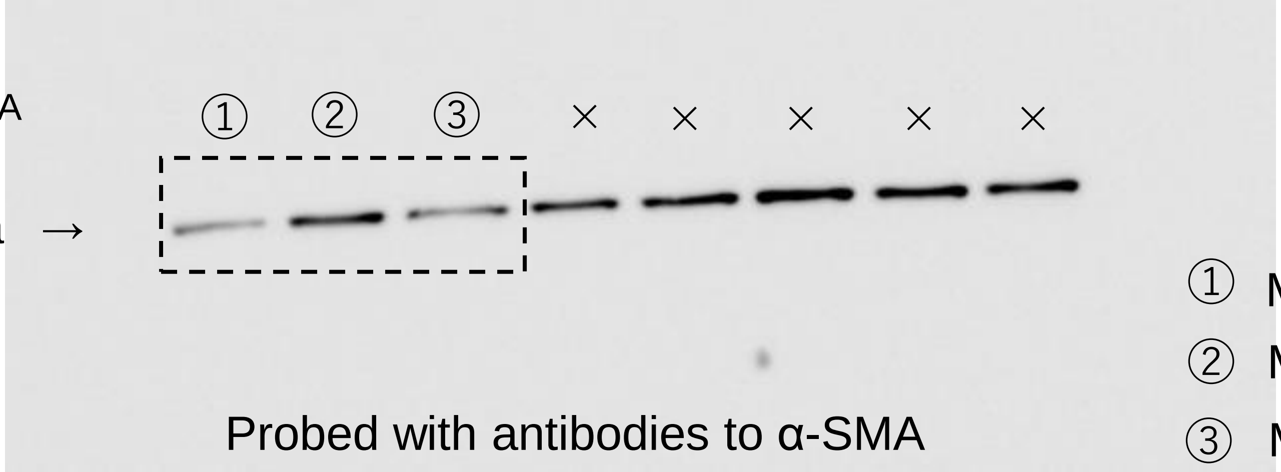


① Mannitol 0 mM
② Mannitol 200 mM
③ Mannitol 200 mM + Y-27632

The band corresponding to GAPDH is indicated by the arrow.

37 kDa →

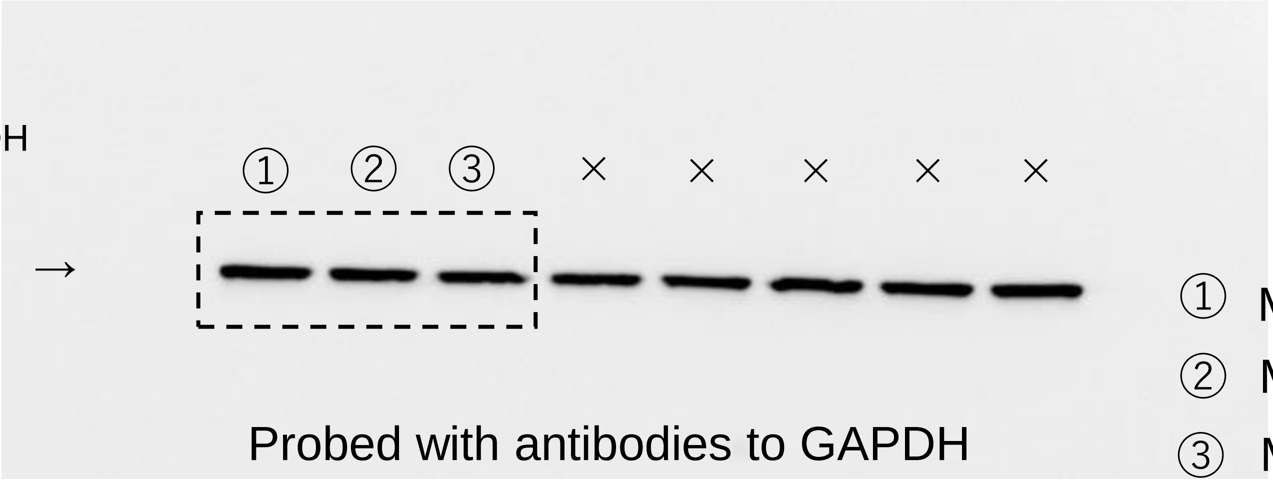


① Mannitol 0 mM
② Mannitol 200 mM
③ Mannitol 200 mM + Y-27632

# S1 raw images

**<u>Original blots from Fig 6A (E-cadherin and GAPDH)</u>**
NRK-52E cells were treated with mannitol (200 mM) or cotreated with Y- 27632 (1 μM) for 12 h.

The band corresponding to E-cadherin is indicated by the arrow.

× ① ② ③ × ×

135 kDa →

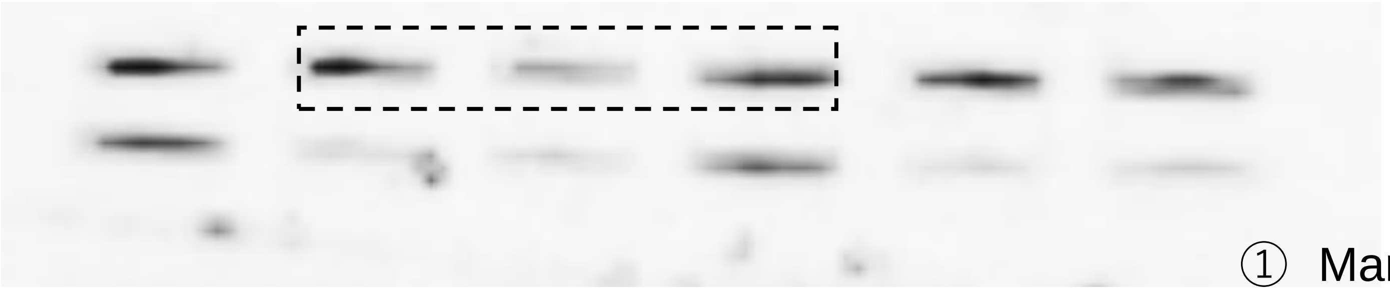

Probed with antibodies to E-cadherin

① Mannitol 0 mM
② Mannitol 200 mM
③ Mannitol 200 mM + Y-27632

① ② ③ × × × × ×

The band corresponding to GAPDH is indicated by the arrow.

37 kDa →

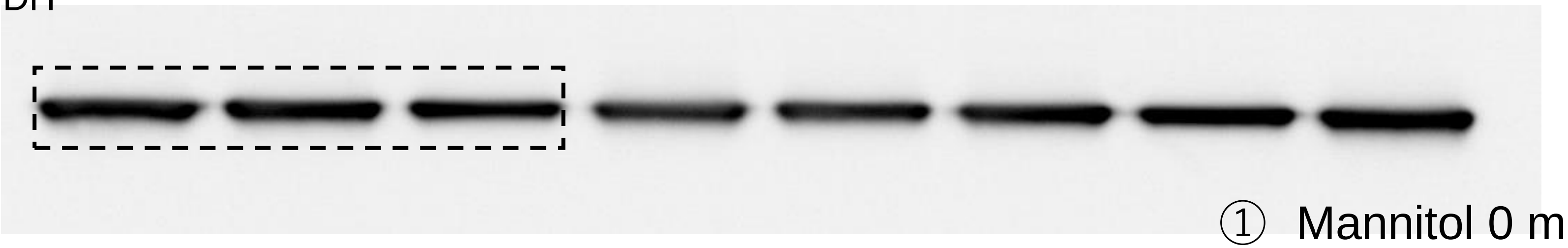

Probed with antibodies to GAPDH

① Mannitol 0 mM
② Mannitol 200 mM
③ Mannitol 200 mM + Y-27632
